# Movement cognition and narration of the emotions treatment versus standard speech therapy in the treatment of children with borderline intellectual functioning: a randomized controlled trial

**DOI:** 10.1186/s12888-017-1309-z

**Published:** 2017-04-20

**Authors:** V Blasi, G Baglio, F Baglio, MP Canevini, M Zanette

**Affiliations:** 1 IRCCS, Fondazione don Carlo Gnocchi ONLUS, Via Capecelatro 66, 20148 Milan, Italy; 2 Department of Health Sciences, University of Milan, Milan, Italy; ASST S. Paolo and S. Carlo Hospital, Milan, Italy

**Keywords:** Borderline intellectual functioning, Development, Movement, Cognition, Narration of emotions, Speech therapy, Rehabilitation

## Abstract

**Background:**

Borderline intellectual functioning (BIF) is defined as a “health meta-condition… characterized by various cognitive dysfunctions associated with an intellectual quotient (IQ) between 71 and 85 which determines a deficit in the individual’s functioning both in the restriction of activities and in the limitation of social participation”. It can be caused by many factors, including a disadvantaged background and prematurity. BIF affects 7–12% of primary school children that show academic difficulties due to poor executive functioning. In many children with BIF, language, movement and social abilities are also affected, making it difficult to take part in daily activities. Dropping out of school and psychological afflictions such as anxiety and depression are common in children with BIF.

This study investigates whether an intensive rehabilitation program that involves all of the areas affected in children with BIF (Movement, Cognition and Narration of emotions, MCNT) is more effective than Standard Speech Therapy (SST).

**Methods:**

This is a multicenter interventional single blind randomized controlled study. Children aged between 6 to 11 years who attend a mainstream primary school and have multiple learning difficulties, behavioral problems and an IQ ranging between 85 to 70 have been enrolled. Participants are randomly allocated to one of three groups. The first group receives individual treatment with SST for 45 min, twice a week for 9 months. The second group receives the experimental treatment MCNT for 3 h per day, 5 days/ week for 9 months and children work in small groups. The third group consists of children on a waiting list for the SST for nine months.

**Discussion:**

BIF is a very frequent condition with no ad hoc treatment. Over the long term, there is a high risk to develop psychiatric disorders in adulthood. Due to its high social impact, we consider it very important to intervene during childhood so as to intercept the remarkable plasticity of the developing brain.

**Trial registration:**

“Study Let them grow: A new intensive and multimodal Treatment for children with borderline intellectual functioning based on Movement, Cognition and Narration of emotions”, retrospectively registered in ISRCTN Register with ISRCTN81710297 at 2017–01-09.

## Background

Borderline Intellectual Functioning (BIF) is a complex clinical entity that has received poor scientific investigation [1, 2]. According to the CONFIL consensus group [3], BIF can be defined as a “*health meta-condition … characterized by various cognitive dysfunctions associated with an intellectual quotient (IQ) between 71 and 85 which determines a deficit in the individual’s functioning both in the restriction of activities and in the limitation of social participation*”. Excluding BIF due to genetic and chromosomal abnormalities syndromes, the mostly frequent causes related to this condition are biological factors, like perinatal complications, and epigenetic factors related to the social environment such as maltreatment, abuse, neglect and high level of maternal stress [1, 4–7]. It is estimated that the prevalence of “non genetic” BIF ranges between 7 to 12% [2, 3] but the lack of terminological consensus and specific diagnostic code in the DSM-V [8] and ICD10 [9] makes it difficult to have a precise estimate. In DSM-V IQ test scores are removed from the diagnostic description and BIF is mentioned only within V-codes; this makes the BIF condition more difficult to be defined and acknowledged [2, 10].

BIF is associated with several neurodevelopmental disorders, with a high frequency for anxiety and behavioral disorders [1, 2, 11–14]. A strong association between BIF and mental disorders is observed in adolescence and in adulthood (e.g. antisocial personality disorder, depression, psychosis, suicide and substance abuse) [12, 15, 16]. The comorbidity with mental disorders is of great relevance in the prognosis: presence of BIF adds further severity to the evolution of psychiatric diseases [17]. Moreover, BIF is a lifelong condition with obvious drawbacks in terms of social and vocational opportunities and health outcomes [1, 13, 15, 18]. A longitudinal study of the impact that mild intellectual deficits, including BIF, have on the life course demonstrated that low-IQ individuals, compared to their higher IQ siblings, completed less schooling, had less prestigious occupations, rated themselves less physically healthy, and reported lower levels of psychological well-being [19].

Furthermore, in childhood, BIF is associated with some degree of motor skills limitation and cognitive difficulties in gaining and generalizing knowledge, in executive functions (i.e. working memory, problem solving, attention and concentration, planning, and inhibition of impulsive responses) and text comprehension [3, 20, 21]. Indeed, children with BIF have a high rate of school failures [18, 22, 23].

Finally, in a recent study [24], we found that children of 7 to 10 years of age with BIF show a delay in the development of social competences. Specifically, they perform poorly on all types of false belief tasks that test the Theory of Mind. Their performance was comparable to children of a younger age (3 to 4 years) who understand that actions are driven by desires, thought, intention and true belief but do not comprehend that behavior can also be justified by misinterpretation, i.e., false belief. These difficulties can result in poor social relations and social disadvantage in adulthood.

Moreover, in a recent study [25] concerning brain morphometry in children with BIF, we detected a delayed development of the parahippocampal, temporal and sensory-motor cortices, important for behavioral, learning and motor abilities. Such delay was highly correlated with IQ indices. Considering the great amount of cerebral modifications that occur in the typical developing brain between 6 to 12 years of age [26–29], we believe that an early and effective intervention is essential to improve the clinical course and the brain development trajectory of children with BIF.

For this reason, we developed an innovative treatment for BIF that stems from three main theoretical considerations: 1- Intelligence is not a fixed entity but a multidimensional and dynamic process; 2- the development of emotional, cognitive and motor skills is interrelated; 3-. environmental enrichment (EE) has a high impact on the neurodevelopment.

In relation to the first point, we considered the definition of intelligence of the Mainstream Science on Intelligence [30], “*It is a very general mental capability that, among other things, involves the ability to reason, plan, solve problems, think abstractly, comprehend complex ideas, learn quickly and learn from experience*”. According to this definition, intelligence can be considered a modifiable entity that is largely dependent on educational, cultural, socioeconomic, emotional and motivational factors. This notion is supported by both behavioral and neuroimaging data [31] showing that individual’s intellectual capacity can decrease or increase in the teenage years and also correlate with independently measured changes in brain structure of specific regions. We consider intelligence important for the development of truly adaptive abilities that ultimately represents a protective factor from social disadvantage in adulthood.

Point 2 refers to recent findings demonstrating that the level of performance in motor, executive functioning and language skills is highly correlated in both typical development [32–37] and in children with BIF [38, 39]. Moreover, the development of the brain areas involved in these functions is closely interrelated [40].

Finally, the third point is related to the well known effect of EE on neurodevelopment: richer and more stimulating environments determine higher rates of synaptogenesis and more complex dendritic arbors, leading to increased brain activity. EE was originally demonstrated in mouse brain [41–43] and successively confirmed on humans [44–47]. Research in humans has shown that attaining and engaging in higher levels of education and living in cognitively stimulating environments results in greater cognitive reserve [48]. The effects of EE take place primarily during neurodevelopment, but also throughout adulthood to a lesser degree [49, 50]. In humans, the opposite phenomenon has also been demonstrated: lack of stimulation in childhood, a critical period for the neurodevelopment, causes delay and impairment in the cognitive development, as in the case of children growing in Institutions that offer impoverished stimulation [51]. Similarly, we consider important, for the rehabilitation of children with BIF, to include different and multiple levels of stimulation as specified in the Methods section. Moreover, we consider part of the EE the capacity to listen and understand in an empathic way the children, focusing not only on their performances but also on their needs and emotions promoting the use of creative strategies to learn instead of fixed predetermined instructions.

All the above-mentioned considerations have been crucial for our choice of a treatment for the BIF. We introduce an innovative, multimodal and intensive training called the Movement Cognition and Narration of emotions Treatment (MCNT). No specific rehabilitation approach is available at the moment for children with BIF, but due to their poor academic performances they are typically treated with Standard Speech Therapy (SST). The latter is aimed at training specific functions such as writing or reading, and/or with the prescription to use facilitation devices such as the calculator and/or the computer.

The aim of our trial was to determine if the MCNT is more effective than SST in improving global intelligence in children with BIF. Our prediction is that MCNT will prove to be more effective than SST due to its use of a multimodal, empathic and game oriented approach, with the use of interactive technology devices, and a great variety of activities related to cognition, psychomotricity and emotions. The difficulties encountered by BIF children, indeed, cannot be delimited to a single specific domain. Consequently, we consider the SST approach as insufficient in treating a complex condition such as BIF. Furthermore, we predict that children with BIF attending the MCNT treatment will show a greater improvement also in the cognitive abilities and in particular, executive functioning. Finally, we will investigate if the MCNT improves the developmental trajectories of brain regions that we previously identified as having delayed maturation in children with BIF: the parahippocampal, temporal and sensory-motor cortices.

## Methods

### Study design

This is a multicentre interventional single blind randomized controlled study with three groups of children with BIF: group 1- children treated with SST (conventional treatment; *N* = 20); group 2-children treated with the MCNT (experimental treatment, *N* = 20); group 3-children not treated -on the waiting list for SST (no treatment *N* = 20).

The Study was approved by the Ethics Committee of the Don Gnocchi Foundation and of the ASST S. Paolo and S. Carlo Hospital.

All parents signed a written informed consent at the first meeting.

**Fig. 1:**
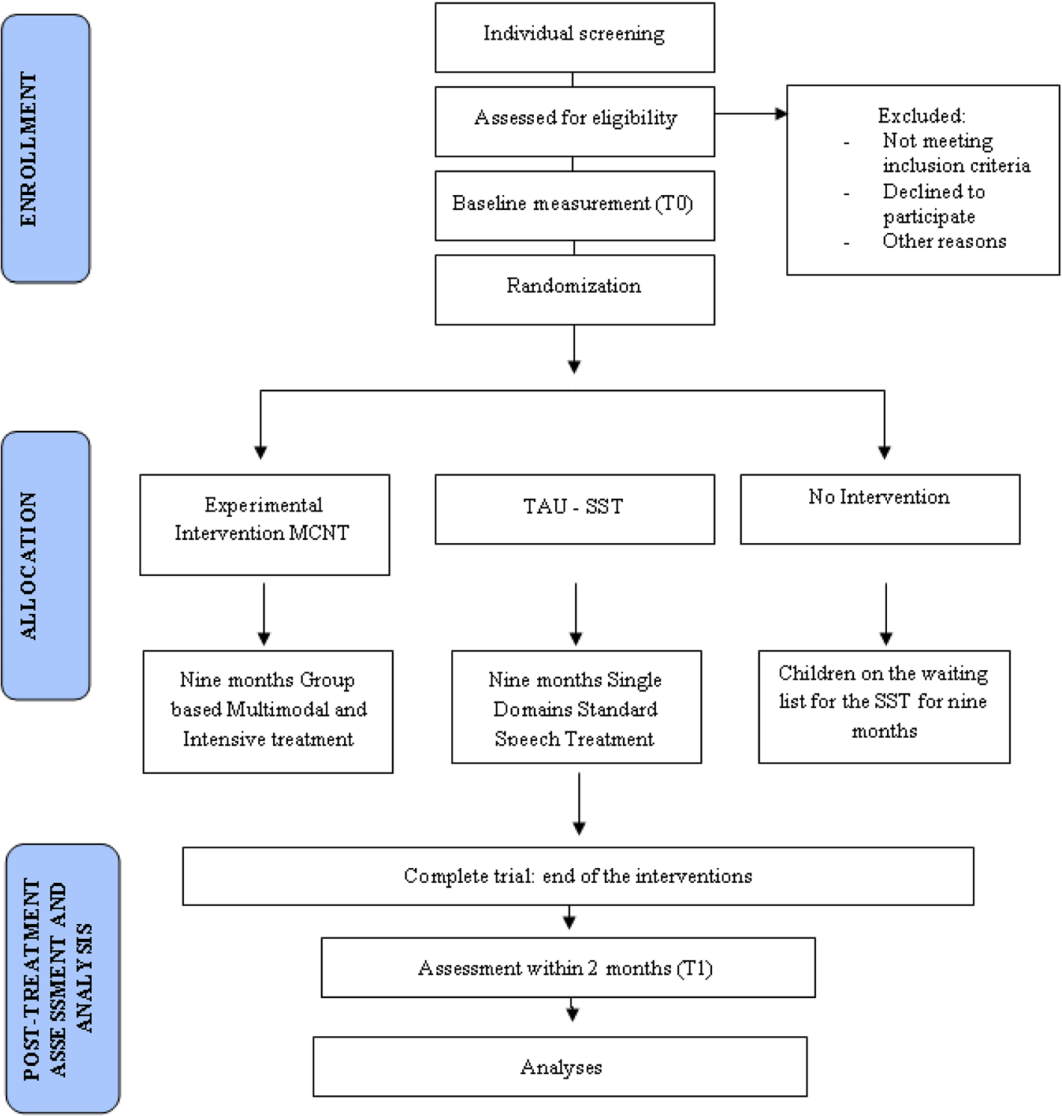
Consolidated Standards Of Reporting Trial (CONSORT) guidelines flow diagram for enrollment and randomization

The primary and secondary outcome measures are determined at two time points, within two months prior to the beginning of the treatment (T0) and within two months after the end of the treatment (T1). Evaluations of all children made before and after treatment are performed by two psychologists blind to the type of intervention that the child receives. The flow diagram for study enrollment and randomization, as per Consolidated Standards Of Reporting Trial (CONSORT) guidelines, is shown in Fig. 1.

**Fig. 2:**
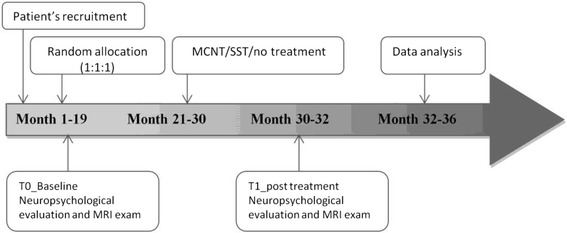
Timeline of the trial. Timing and duration of the various procedures used in the trial

### Sample size

For the power calculation, considering preliminary data from our group, we expect a delta of 8 points (T1 –T0) in the primary outcome measure (WISC III FSIQ) between SST and MCNT groups, and 10 points between MCNT and no treatment group with a standard deviation of 10. With a sample size of 60, the calculation of the effect size is 0.43 and the probability is 0.83.

Standard procedures to maintain participants’ interest in the study and to avoid drop outs will be applied (regular meetings with teachers and parents of the children). Moreover, since the MCNT requires children to attend the treatment four day/week, we organized a shuttle that brings them from their school to our Centre brings, to avoid drop-outs.

### Participants

Sixty participants will be consecutively recruited from the Child and Adolescent Neuropsychiatry Unit of IRCCS Don Carlo Gnocchi Foundation and of the ASST S. Paolo and S. Carlo Hospital.

Inclusion criteria are: age range between 6 to 11 years old and attending primary mainstream school; with a Full Scale Intelligence Quotient (FSIQ) score ranging from 70 to 85 determined with the Wechsler Intelligence Scale for Children-III (WISC-III) [52, 53]; presence of learning disabilities assessed with the standardized test battery for developmental dyslexia and dysorthographia (DDE-2) [54] and dyscalculia (AC-MT 6–11) [55]; difficulties in executive functions and or verbal comprehension assessed with Modified Barrage bell test (MBT) [56],Tower of London (TOL) [57, 58], and the Test of Reception of Grammar (TROG 2) [59, 60]; presence of an impact on daily life of the above mentioned difficulties as measured by the Child Behavioral Checklist (CBCL 6–18) [61, 62] and Vineland 2 [63].

The exclusion criteria are: presence of major neuropsychiatric disorders (such as ADHD and autism spectrum disorder); presence of neurological conditions such as epilepsy, traumatic brain injury, brain malformations and infectious disease involving the central nervous system. Moreover, the presence of systemic diseases such as diabetes or dysimmune disorders, genetic syndromes such as Down syndrome or Fragile X syndrome and a positive history for psychoactive drugs, particularly referring to current or past use of psychostimulants, neuroleptics, antidepressants, benzodiazepines and antiepileptic drugs are also considered exclusion criteria.

### Randomization and masking

Randomization in the three groups occurred after screening and baseline assessment (T0). Subjects were randomly assigned to SST or MCNT or no treatment. The randomization process was performed using a computer algorithm (http://graphpad.com/quickcalcs/randMenu/) by an independent operator not involved in neither phase of our research. All evaluations in both pre- and post-treatment are conducted by two psychologists blind to group allocation.

### Intervention

Our study involves two types of intervention: the MCNT, which represents the experimental intervention; and the SST, the treatment as usual (TAU). Both treatments last for nine months and provide regular meetings between the professionals responsible for the treatment and the families and the teachers of the children. Regular weekly meetings among professionals are also included in both treatments. The MCNT is based on a multidimensional approach with children working in small groups while the SST is domain specific and treatment is one-on-one based.

#### Experimental treatment: The movement cognition and narration of the emotions treatment (MCNT)

The MCNT is an intervention in which children work in small groups, for 3 h each day, five days a week, Monday through Friday, for nine months. Each day they arrive at 2 pm, with a shuttle that brings them from their school to our Centre. They are divided into 3 groups, for the whole treatment, named Red, Blue and Green, according to their global functioning, grade and/or special educational needs. Upon arrival children are gathered all together for a 15 min long relaxing activity, after which each group starts with one of the three laboratories for 45 min. At the end of the first, the three groups switch the activity until all groups have completed all three laboratories.

MCNT is focused on the three domains affected in children with BIF that are strictly embedded with learning abilities: motor, cognitive and socio-emotional domain. This treatment consists of: a) Movement training to improve motor planning and fine and gross motor abilities with a Game Therapy approach using the Wii and Xbox video game platforms; b) Cognitive training for the empowerment of the executive functions such as working memory, planning abilities, problem solving, reasoning with the use of the multimedia interactive whiteboard (MIW); and c) an emotive training to learn how to Narrate the emotions to help the child to cope with the experiences of her/his daily life.

We chose to use the technology devices mentioned above because they are able to capture the attention and are highly motivating for the child. In addition, the use of the MIW represents a very useful visual aid for a visually-oriented teaching strategy that is effective in fostering verbal comprehension, memory encoding and storage processes. With both devices, moreover, it is possible to promote small competitions between the children which are very effective in motivating the child and improving information speed processing, positive interdependency and frustration tolerance. Finally, to help the child to narrate the emotions, a psychotherapist promotes several activities such as the narration of fairy tales, or drawing and symbolic playing to create a new story, to promote the capacity to think, to recognize and to master the emotions.

The first level concerned the movement execution and planning abilities: the objective is to improve balance, fine and gross motor abilities, hand-eye coordination, impulsive motor response inhibition, praxic abilities and attention. For this intervention, the Nintendo Wii Console with the “Wiimote” and the “balance board”, and the “Xbox 360 Kinetic” platforms were used. A game therapy approach is used to improve the children’s motivation and compliance. In the first weeks of treatment, the focus is on easier visual-motor integration skills, and the children familiarize themselves with the Wiimote while using it as a laser pointer to match a fixed visual input on the screen. A second step involves more complex games whereby the child points the Wiimote to a moving target (Wii Motion Plus game) that requires an increased involvement of attention and higher visual-motor integration ability. A third step involves games such as Wii Sports in which children use both the Wiimote and the Wii Balance board to train balance and coordination of both upper and lower limbs. Also, Wii Music and Wii Party games are used to train rhythm, timing of movement and inhibition of impulsive motor behavior. During the whole process, advanced executive functions, such as planning competence, working memory and inhibitory control are involved and trained so as to render the movements more fluid, economical, quicker and functional. Finally, the Xbox Kinetic console is used to train not only all body segments motricity but also moving in unison with another child. For example, in the Kinetic Adventure game, children maneuver a boat on river rapids in pairs with the movements of their bodies. In this step, the child needs to continuously adjust his/her own position in response to the movements of the other person. This activity facilitates training the awareness of his/her own body in space and relative to the other along with the capacity to master a novel situation in a cooperative manner.

The second domain concerns the cognitive abilities. The cognitive training is focused on several domains that include language comprehension and expression and executive functions, such as deductive and inductive reasoning, working memory, planning and problem solving, attention and concentration, inhibition of impulsive verbal responses. This part of the MCNT involves the use of the MIW. Particular attention is devoted to finding appropriate strategies to solve problems and to the constant training of the abilities to increase the velocity with which the task is executed. The aim is to promote the ability to explore the information, to select salient features which are relevant to solve the problem and finally to take a decision while examining the possible choices. Moreover, children are trained at keeping and sharing their attention with all members of the group for the whole duration of the session by promoting their active participation in the activities. Finally, according to the incremental intelligence theory [64], children are supported in the idea that their intelligence and their capacities can be increased by training and that each child has a different way to learn and to encode sensorial stimuli: some rely more upon visual strategies while others prefer verbal modalities. Indeed, great attention is devoted to the cognitive style and the learning strategies of each child. This approach helps the child to focus on the discovery of his/her personal functioning rather than being worried about the grade. Finally, working in a group promotes prosocial behavior and cooperative learning.

The third domain concerns emotions and social skills. BIF children very often lack adaptive skills and show behavioral, emotional and/or social-relations problems. The focus of this part of the treatment is to help the children to express, recognize and cope with their own emotions. We named this process “alphabetization” of the emotions [65]. The underlying idea stems from the psychoanalytic model of Bion [66, 67] in which the comprehension of the emotional experience is central to thought and learning. The term “alphabetization” refers to the capacity to transform the unknown sensations that precede and accompany any emotion into elements that can be thought and narrated [66, 67]. This process demands that the unknown sensations, which can generate discomfort, can be tolerated and contained in the mind of the subject and/or of the therapist for a sufficient time to be transformed into symbols that can be thought, experienced and communicated [65]. The alphabetization process operates through a symbolization mechanism that implies the “as if” process. Giving a simple name to the emotion is not sufficient, it is necessary to transform it into a metaphor, a story or a play. Such a process is very important for social relations: if the child can express his/her inner states, he/she has no need to act rashly upon them. Finally, the alphabetization process helps the child to “clear” the mind from very unwanted thoughts and sensations, which can ultimately facilitate the cognitive processes. The therapist, a psychologist with a psychotherapy degree, uses different approaches to promote the narration of the emotions: symbolic play, reading, inventing and/or dramatizing a story, drawing and talking. These activities are considered creative processes with a transitional function [68] that enable the children and the therapist to deal with the emotions in an indirect way, preventing the fear of being judged and persecutory feelings. Finally, such a transitional space enables and promotes the “as if” process [68]. The therapist helps the children to associate the feelings and emotions narrated and symbolized through the activities with their own experiences. Within the group, each child can introduce new characters, new scenarios and/or situations to challenge the group to find solutions. The goal is to encourage cooperation and to experience the facilitation effect of the group in thinking and coping with emotions.

#### Treatment as usual: The standard speech therapy (SST)

STT is an individual treatment consisting of two 45 min sessions each per week for nine months. The focus is on the training of the academic abilities that are compromised in the child as assessed by the neuropsychological evaluation at T0 (pre-treatment). Typically, the skills mostly affected are reading comprehension, word-meaning relationship (semantics); writing problems related to phoneme-grapheme correspondence; arithmetic abilities; and problem solving. To empower these skills, SST uses both pencil/paper tools and specific rehabilitation software (http://www.erickson.it/). In the event of dyslexia or dysorthographia, the principal aims of SST are to increase processing information speed and transcoding, reducing spelling mistakes and expanding personal vocabulary. One of the approaches used is sub-lexical treatment. For dyscalculia, images are used to reason and solve the problem as, for example, in the analogical method [69]. Moreover, the empowerment of transversal competences such as phonological competences, perception, visual-spatial ability, attention, memory and other executive functions is pursued together with the use of compensative tools. Key criteria are functional re-education and the use of a meta-cognitive approach. The first provides for using alternative/different strategies to cover or partially rehabilitate compromised mechanisms whereas the second is based on strategies to focus the attention on mental processing (input, elaboration and output). Finally, the speech therapist offers pedagogy consultation for both parents and teachers to increase their knowledge concerning learning difficulties and potentially associated behavioral-emotional problems and finally to share effective problem solving strategies.

#### Assessment design and outcomes measures

The timeline of the study is illustrated in Fig. 2. All children are evaluated at two time points, within two months prior to the beginning of the treatment (T0) and within two months after the end of the treatment (T1). For the no-treatment group the same time interval is kept.

To measure intellectual functioning and motor competences, we used the WISC-III [52, 53] and the Movement Assessment Battery for Children (M-ABC) [70], respectively.

The Emotional Quotient Inventory-Youth Version (EQ-i:YV) [71] was used to assess the emotion competences, while the Child Behavior Check List (CBCL 6–18) [61, 62] and Vineland 2 [63] were employed for investigating adaptive behavior.

A full neuropsychological evaluation was also made with the use of following tests: the Modified Bells Test (selective attention MBT) [56]; the Tower of London (TOL) planning ability and inhibitory control; [57, 58]; the Neuropsychological Evaluation Battery for developmental age 5–11 (BVN5–11, speech fluency, selective word retrieval and Corsi) [72] to assess verbal executive function and verbal and visual short term memory; the Test of Reception of Grammar-2 (TROG2, language comprehension) [59, 60].

Finally, our study includes measures of brain morphometry and function, evaluated by means of magnetic resonance imaging (MRI) as surrogate outcome measure. All children participating in the study are evaluated with non-conventional brain MRI to investigate brain morphometry and functionality in the regions that we found abnormally developed in our previous work [25]: the parahippocampal, temporal and sensory-motor cortices.

#### Outcomes

Primary and secondary outcome are evaluated at T0 and T1.

The primary outcome measures are:1 WISC-III; 2. M-ABC; 3. EQ-i:YV; 4. CBCL 6–18 and Vineland 2.

Secondary outcome measure are: 1. MBT; 2. TOL; 3. BVN5–11; 4. TROG2; and MRI.

### Analysis

Statistical analyses on outcome measures will be conducted using R and SPSS. The General Linear Model (GLM) will be used to test the interaction effects between treatment (MCNT vs no treatment, MCNT vs TAU, TAU vs no treatment) and time (2 repeated measures, T0 and T1). Violations of the parametric model assumptions will be tested and explored with ad-hoc techniques. If violations will be detected, robust methods, data manipulation or statistical corrections will be applied accordingly. An intention-to-treat analysis approach will be used. The missing data process will be examined and the proper imputation method will be selected thereafter.

MRI data will be analyzed with specific statistical software: SPM12, (http://www.fil.ion.ucl.ac.uk/spm/); and FreeSurfer (https://surfer.nmr.mgh.harvard.edu/) and FSL (https://fsl.fmrib.ox.ac.uk/fsl/fslwiki/). Random effect analyses will be done to detect if there is an effect of treatment on the variables measured. The model will comprise a between factor level (group) and a within factor level (time). Demographic characteristics will be used as covariates if associated with the outcome measure.

## Discussion

Borderline Intellectual Functioning is a very frequent condition with a high social impact due to poor school achievements and long term high risk to develop psychiatric conditions in adulthood.

In our protocol, we aim to measure the efficacy of an intensive training that, contrary to the TAU, works in parallel on all domains affected in borderline intellectual functioning: movement, cognition and emotions.

As mentioned in the introduction, it is important to intervene during childhood so as to intercept the remarkable plasticity of the developing brain in which dendrites and spines are able to form new synapses in hours and even minutes in response to some experiences [73]. This holds true for both positive and negative experiences, such as effective rehabilitation programs and adverse events, respectively. The last, when occurring early in life, can produce long term modifications in brain functioning associated with a “latent vulnerability” for psychiatric disorders in adulthood [74]. Recent studies, indeed, have consistently shown hyperactivity in the amygdala when children that have been exposed to early adversity or maltreatment are stimulated with threat-related cues [75] [76].

According to these premises, we claim the importance of finding an effective treatment for children with BIF, which is capable of intercepting the great potentiality of the developing brain and to realign the development towards more typical trajectories.

### Trial status

The trial is ongoing.

## Abbreviations

ADHD: Attention Deficit Hyperactivity Disorder
BIF: Borderline Intellectual Functioning
BVN: Neuropsychological Evaluation Battery for developmental age
CBCL: Child Behavior Check List
EQ-i:YV: The Emotional Quotient Inventory-Youth Version
M-ABC: Movement Assessment Battery for Children
MBT: Modified Bells Test
MCNT: Movement Cognition and Narration of the emotions Treatment
MRI: Magnetic Resonance Imaging
SST: Standard Speech Treatment
TAU: Treatment As Usual
TOL: Tower Of London
TROG: Test for Reception Of Grammar
WISC-III: Wechsler Intelligence Scale for Children
